# The Costs of Respiratory Illnesses Arising from Florida Gulf Coast *Karenia brevis* Blooms

**DOI:** 10.1289/ehp.0900645

**Published:** 2009-05-01

**Authors:** Porter Hoagland, Di Jin, Lara Y. Polansky, Barbara Kirkpatrick, Gary Kirkpatrick, Lora E. Fleming, Andrew Reich, Sharon M. Watkins, Steven G. Ullmann, Lorraine C. Backer

**Affiliations:** 1 Marine Policy Center, Woods Hole Oceanographic Institution, Woods Hole, Massachusetts, USA; 2 Environmental Health Program, Mote Marine Laboratory, Sarasota, Florida, USA; 3 Miller School of Medicine and Rosenstiel School of Marine and Atmospheric Sciences, University of Miami, Miami, Florida, USA; 4 Aquatic Toxins Program, Bureau of Community Environmental Health, Florida Department of Health, Tallahassee, Florida, USA; 5 Department of Management, University of Miami, Miami, Florida; 6 National Center for Environmental Health, Centers for Disease Control and Prevention, Atlanta, Georgia, USA

**Keywords:** cost of illness, emergency department (ED), harmful algal bloom (HAB), economic impact, natural hazard

## Abstract

**Background:**

Algal blooms of *Karenia brevis*, a harmful marine algae, occur almost annually off the west coast of Florida. At high concentrations, *K. brevis* blooms can cause harm through the release of potent toxins, known as brevetoxins, to the atmosphere. Epidemiologic studies suggest that aerosolized brevetoxins are linked to respiratory illnesses in humans.

**Objectives:**

We hypothesized a relationship between *K. brevis* blooms and respiratory illness visits to hospital emergency departments (EDs) while controlling for environmental factors, disease, and tourism. We sought to use this relationship to estimate the costs of illness associated with aerosolized brevetoxins.

**Methods:**

We developed a statistical exposure–response model to express hypotheses about the relationship between respiratory illnesses and bloom events. We estimated the model with data on ED visits, *K. brevis* cell densities, and measures of pollen, pollutants, respiratory disease, and intra-annual population changes.

**Results:**

We found that lagged *K. brevis* cell counts, low air temperatures, influenza outbreaks, high pollen counts, and tourist visits helped explain the number of respiratory-specific ED diagnoses. The capitalized estimated marginal costs of illness for ED respiratory illnesses associated with *K. brevis* blooms in Sarasota County, Florida, alone ranged from $0.5 to $4 million, depending on bloom severity.

**Conclusions:**

Blooms of *K. brevis* lead to significant economic impacts. The costs of illness of ED visits are a conservative estimate of the total economic impacts. It will become increasingly necessary to understand the scale of the economic losses associated with *K. brevis* blooms to make rational choices about appropriate mitigation.

Harmful algal blooms (HABs) of *Karenia brevis*, a marine dinoflagellate, occur almost annually in the Gulf of Mexico off the west coast of Florida (Heil and Steidinger 2009). *K. brevis* cells produce potent polyether neurotoxins known as brevetoxins (Baden et al. 1995; Poli et al. 1986), which are released into the ocean when the cells are lysed by wind and waves (Pierce et al. 2001). At high concentrations, *K. brevis* blooms may cause mortality of fish, marine mammals, and sea birds. Humans who consume shellfish contaminated with brevetoxins are at high risk of developing neurotoxic shellfish poisoning (NSP) (Watkins et al. 2008). Further, there have been anecdotal reports of skin ailments resulting from contact with brevetoxin-contaminated water (Kirkpatrick et al. 2004).

HAB cell count generally correlates with brevetoxin concentration, and residual brevetoxins in seawater have been observed after blooms have diminished (Pierce RH, personal communication). Bubbles from breaking waves transport brevetoxins to the sea surface, where they may be released to the air as jet drops when the bubbles burst (Blanchard 1989; Pierce et al. 1990, 2003). In the air, brevetoxins become incorporated into marine aerosols, which are built around charged salt particles. Aerosol transport is highly influenced by wind speed and direction, and aerosolized brevetoxins can travel as much as 1 mile inland (Fleming et al. 2005; Kirkpatrick et al. 2008).

Woodcock (1948) reported the first accounts of respiratory irritation in humans during severe red tide (HAB) conditions on the Florida Gulf Coast. Subsequent studies have linked inhalation of aerosolized brevetoxins with adverse health effects, including rhinorrhea, nonproductive cough, and severe bronchoconstriction (Asai et al. 1982; Backer et al. 2003; Cheng et al. 2005; Fleming et al. 2005, 2007; Kirkpatrick et al. 2004, 2001; Music et al. 1973). Epidemiologic studies, animal experiments, and anecdotal reports now suggest that aerosolized brevetoxins are linked to both upper and lower respiratory illnesses in humans (Backer et al. 2003; Fleming et al. 2005, 2007; Kirkpatrick et al. 2004). In addition to significantly increased respiratory symptoms, asthmatic individuals have shown small but statistically significant changes in lung function immediately after 1-hr visits to the beach during Florida blooms (Backer and McGillicuddy 2006; Fleming et al. 2005, 2007). After only 1 hr of exposure to aerosols at the beach, respiratory complaints may last up to 5 days in asthmatics (Kirkpatrick et al. 2008). Finally, research using data for respiratory visits to the emergency department (ED) demonstrated a significantly increased risk of visits for pneumonia, bronchitis, and asthma during a Florida red tide period compared with a similar period without red tide (Kirkpatrick et al. 2006). This risk is particularly relevant for coastal residents.

In the present study we assessed the relationship between *K. brevis* blooms and respiratory illness-related visits to hospital EDs while controlling for environmental factors and disease that also may function as significant risk factors for respiratory ailments. Further, we developed estimates of the costs of illness resulting from medical treatments for acute brevetoxin-related respiratory illnesses. This work is an important step toward understanding one component of the economic effects of *K. brevis* blooms. It may help guide the selection and implementation of management actions to mitigate public health effects and illness costs for brevetoxins and possibly other HABs. Further, it may provide insight to health care administrators and providers as to human resource and medical supply needs as a result of *K. brevis* blooms.

## Materials and Methods

### Methods

Aerosolized brevetoxins may result in respiratory illnesses of varying severities. Many individuals with respiratory symptoms caused by brevetoxins may respond initially by purchasing over-the-counter medicines or taking time off from work or leisure. At present, data on visits to physicians and out patient facilities are unavailable for unreported disease. For low-severity illnesses, medical costs are uncertain but are expected to be minor and unlikely to involve significant productivity losses. In this study, we focused on visits to EDs for which we have credible data. There also may be cases of further hospitalizations or mortalities, but we expect that the number of patients experiencing these severities is small. In particular, we are unaware of any deaths caused by aerosolized brevetoxins.

We focused on the relationship between HAB cell counts and respiratory hospital ED visits from 2001 to 2006 in Sarasota, Florida. Sarasota experiences nearshore *K. brevis* blooms on an almost annual basis; in some years, there are multiple or even continuous blooms. These events have been documented through monitoring efforts by researchers at Mote Marine Laboratory (MML). We focused on Sarasota Memorial Hospital (SMH), the hospital located closest to the coastline of the four hospitals in Sarasota County. SMH is the largest acute-care facility in Sarasota County, serving 63.3% of the county’s population (Kirkpatrick et al. 2006).

In our analysis we concentrated on two main types of respiratory conditions: all respiratory diseases taken together and a combination of upper airway disease (UAD) and chronic/acute bronchitis. Previous work by Kirkpatrick et al. (2006) demonstrated that UAD and bronchitis (as well as pneumonia and asthma) are particularly likely to be exacerbated by aerosolized brevetoxins. Adverse respiratory conditions may be attributable to HABs or a combination of HABs with other ambient factors.

We developed an exposure–response model to express hypotheses about the relationship between respiratory illnesses, HAB events, and other potential explanatory variables. The model is formulated as follows:


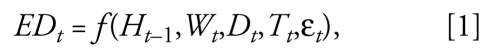


where the subscript *t* indexes the relevant week; *ED* is a measure of the number of respiratory illness ED visits at SMH; *H* is a lagged measure of *in situ K. brevis* cell counts near the Sarasota coast; *W* is a vector of environmental and weather conditions; *D* is a measure of regional respiratory disease outbreaks; and *T* is a measure of tourist visits to Sarasota County. Equation 1 is estimated using autoregressive error models.

### Data

We investigated a large number of environmental, disease, and tourism variables (Polansky et al. 2007). We present two parsimonious models here. Variables investigated but not included in the final model specifications are mentioned briefly below. Descriptive statistics for model variables appear in Table 1.

The total number of daily SMH ED visits for respiratory diagnoses were compiled from October 2001 through September 2006. Access to anonymous medical data was provided by SMH after its Institutional Review Board’s approval of the use of data for our study. Using codes from the *International Classification of Diseases, Ninth Revision, Clinical Modification* (ICD-9-CM), these diagnoses were categorized as respiratory illnesses (e.g., pneumonia, bronchitis, asthma, UAD) or all other primary diagnoses (NCHS 2009). We calculated for each week the daily average number of ED visits for two categories of respiratory diagnoses (all four diseases and a combined UAD/bronchitis).

*In situ K. brevis* cell counts served as a proxy for aerosolized brevetoxin concentrations along the coast. Cheng et al. (2005) developed an empirical relationship between brevetoxin concentration in the ocean and in the atmosphere as a function of wind speed and wind direction. Aerosolized brevetoxins have been measured as much as 1 mile inland from the coast (Kirkpatrick et al. 2008); thus, the affected population may include those who are not right on the coast or do not visit the beach or shoreline. Measurements of brevetoxin concentrations in the ocean are time consuming and costly to make. Consequently, long-term brevetoxin concentration data sets are unavailable. We assumed that because higher HAB cell counts would yield increased brevetoxin concentrations in water, and subsequently in air, they were a reasonable proxy for aerosolized toxin.

*K. brevis* cell counts were sampled at two Sarasota Bay locations [New Pass (27.19°N, 82.34°W) and the MML Bay Dock (27.33°N, 82.58°W)]. Water samples were analyzed weekly during nonbloom conditions and daily during blooms. We averaged the data from both sampling stations to obtain a measure of *in situ* cell count. To create a consistent data set, we compiled for each week the daily average of *K. brevis* cell counts across both sampling stations. Because of the large range of cell counts, we transformed the data for scale purposes. The HAB measure we employed in the model is the square root of one-thousandth of the weekly average of daily average *K. brevis* cell counts.

Pierce et al. (2001) determined that toxins are released into the water as cells rupture, thereby increasing the amount of extracellular toxins as a bloom progresses. After release into marine waters, the toxin subsequently may become aerosolized and transported by winds onshore, where humans may be exposed (Pierce et al. 2003). We expected to find a lag between the detection of cells in offshore waters and onshore aerosolized brevetoxin exposures because of the time required for intra- and extracellular brevetoxin metabolism and transformation, as well as subsequent aeolian transport. A latency period associated with the observed health effects of *K. brevis* blooms has not been established, however. In the model, we tested for a 1-week lag for the *in situ* HAB cell count. We were unable to find correlations between ED visits and measures of wind speed, wind direction, or wind velocity.

Information on air temperature was collected from a monitor at the MML New Pass Weather Station, located at 27.19°N, 82.34°W (MML 2009). We expected lower temperatures would increase the susceptibility of individuals to respiratory illnesses, possibly with a lagged effect. We compiled the data as a weekly average temperature (°C). We were unable to find correlations between ED visits and a measure of relative humidity.

Pollen is a common allergen that can produce histaminergic responses in sensitive individuals, exacerbating existing respiratory conditions or initiating new ones (Andersson et al. 2003; Atkinson and Strachan 2004; Burge and Rogers 2000). Daily pollen counts (pollen grains per cubic meter of air) were provided by the National Allergy Bureau (Jelks M, personal communication). Counts were obtained using a Burkard 7-day pollen collector at the regional sampling station, located approximately 160 miles northeast of Sarasota at the University of Florida in Gainesville. Counts are given as the square root of pollen grains per cubic meter of air × 10^−2^. We also investigated mold counts and local pollution measures (ozone, two sizes of airborne particulates, forest fires, and acres of forest burned). None of these variables were found to correlate with ED visits.

Weekly influenza virus outbreak data for the South Atlantic Region were compiled by the U.S. Centers for Disease Control and Prevention (CDC). These data are a measure of the percentage of specimens testing positive for influenza within a particular week, as measured by the World Health Organization and the National Respiratory and Enteric Virus Surveillance System laboratories. Data were provided from October through May (weeks 40 through 52 or 53 and weeks 1–20), as this time frame is known to correspond to epidemic periods (Simonsen et al. 1997). The CDC does not track influenza during minimal outbreak periods (weeks 21–39). We assumed that these gaps in the data correspond to periods of little or no influenza cases, and we replaced them with zeroes.

We obtained data on Sarasota County monthly hotel/motel occupancy rates for October 2001–September 2006 and the number of units by lodging type (e.g., hotel/motel, campsite, mobile home, apartment, condominium, house) from the Sarasota Convention and Visitors Bureau (Haley V, personal communication). We assumed that the percent occupancy of all lodging types approximates that of hotels/motels. We derived a total number of occupants by lodging type by making the assumptions of two people per hotel/motel, condominium, or apartment unit and four people per campsite, mobile home, or house. We obtained a monthly estimate of the temporary resident population by summing the number of occupants in all units. The tourism variable is measured as persons × 10^−4^. Note that the weekly estimates used in the models remained constant for the weeks within each month.

## Results

### Exposure–response model

Table 2 presents Yule-Walker estimates of two different autoregressive error models corrected for third-order autocorrelation. Each model considers a different dependent variable. In model I, the dependent variable is the natural logarithm of SMH ED visits for all respiratory ailments (lnED_tot_). In model II, the dependent variable is the natural logarithm of SMH ED visits for the combination of UAD/bronchitis (lnED_ub_). Note that the latter measure is included in the former, which also includes ED visits due to pneumonia and asthma.

**Figure 1:**
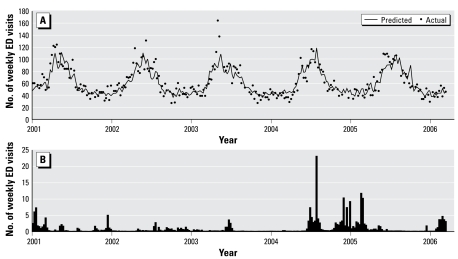
Weekly ED visits for UAD and bronchitis. (*A*) Number of actual SMH ED visits in each week for UAD and bronchitis and the predicted number of visits for the same illnesses using model II. (*B*) Number of ED visits in each week for the same illnesses predicted to have been caused by brevetoxin exposure.

All parameter estimates have the expected signs and are significant at the 10% level or better in both models. The model results show that ED visits for respiratory ailments are correlated with lagged *K. brevis* cell counts, regional pollen counts, regional influenza outbreaks, and Sarasota County tourist visits, and inversely correlated with temperature and temperature lagged 1 week. Both models explained about three-fourths of the variation in their respective dependent variables. Figure 1A illustrates how well the model predicts ED visits compared with the actual data. The marginal effects (the incremental change in ED visits with an incremental change in the variable of interest) and the elasticities (percentage change in ED visits with a percentage change in the variable of interest) are shown in Table 3. Both measures reveal the importance of temperature and tourist numbers as predictors of ED visits.

The inverse relationship between temperature and respiratory ED visits may result from the interaction of other agents responsible for respiratory ailments (e.g., bacteria and viruses). Like influenza, these triggers tend to affect more individuals when low ambient temperatures prevail (Simonsen et al. 1997).

Importantly, our measure of aerosolized brevetoxins is a significant predictor of ED visits for respiratory ailments. Figure 1B depicts the number of SMH UAD/bronchitis ED visits due to *K. brevis* blooms predicted by model II. Because of variable transformations, the parameter value cannot be interpreted per se as the marginal effect of changes in cell counts on ED visits. The marginal effect is itself a function of the cell count, and therefore its value will depend on the state of a *K. brevis* bloom.

In Table 4, we present model predictions of yearly ED visits for three different bloom levels: low, medium, and high. The 4-year period from October 2001 to December 2004 and January 2006 to September 2006 was used for the low-level estimate. The entire 5-year data set from October 2001 to September 2006 was used for the medium-level estimate. The period from January 2005 to December 2005 (a period with a major *K. brevis* bloom) was used for the high-level estimate. We present 5-year predictions of ED visits as well, representing the potential number of cases should bloom levels persist for the relevant level.

Referring to the results of model II in Table 4, the model’s predictions yield annual estimates of the likely number of ED visits for low (39 visits), mean (76 visits), and high (218 visits) bloom levels in Sarasota County. We developed our estimates using data for SMH, which treats 63.3% of the patients in Sarasota County. We have adjusted the estimates for Sarasota County accordingly. These estimates are made holding all other model variables constant at their respective means.

### Cost-of-illness estimates

A measure of the economic impact of respiratory illnesses caused by aerosolized brevetoxins is the sum of the costs of medical services and lost productivities during the illness period. When considering the costs of a specific illness, especially one that does not involve unusual technologies, treatments, or medical skills and one that does not comprise a large portion of the total number of visits, estimates of the marginal costs of treatment are appropriate. In the case of respiratory ailments that are the consequence of exposure to aerosolized brevetoxins, the additional admissions per week (on average only 1.5% of the total) are unlikely to lead to the need to expand capacity in respiratory ED services at SMH.

In many cases, hospitals must charge patients more than average or marginal costs for specific medical services. The excess of charges over costs is generally used to cover the burden to a hospital of the provision of medical services that go unpaid because of the lack of insurance coverage or the inability or unwillingness of patients to pay for treatment. Hospitals often cross-subsidize medical services within their facilities using the net revenues of services with a high inelasticity of demand, such as emergency services, to subsidize those important services that may have a more elastic demand or a lower net revenue margin. Cost-of-illness estimates based on average charges likely exaggerate the true costs of ED visits. In the present study, we applied an estimate of the ratio of marginal to average charges for general ED visits to approximate the marginal costs of ED visits for respiratory ailments due to aerosolized brevetoxins.

Williams (1996) studied the costs of ED visits in six Michigan community hospitals and developed estimates of both average and marginal costs of ED visits. The author estimated the marginal costs of ED visits for three different illness severities: $24 for nonurgent treatments, $67 for semiurgent treatments, and $148 for urgent treatments. Marginal costs averaged only about 23% of average costs.

The Florida Agency for Health Care Administration (AHCA) compiles data on charges levied by Florida hospitals for individual and total medical services for primary diagnosed illnesses. Most of the charges for UAD/bronchitis illnesses involve emergency treatments, radiology services, and laboratory tests. Focusing on the relevant ICD-9-CM codes for UAD/bronchitis illnesses, we calculated the 25th and 75th percentile estimates for the total ED visit charges and found that they ranged from $252 to $1,045 per visit. We applied the Williams (1996) ratio of marginal to average costs of 23% to calculate a range of marginal charges from the AHCA data of $58–$240 as our preferred range of marginal cost estimates.

Where illnesses are severe enough to result in lost time at work or lost leisure opportunities, an estimate of the cost of illness also should include an estimate of lost productivity. We assumed that individuals valued their leisure time at the margin at approximately the same rate as their employment wages. We used annual median personal income in 2006 as a measure of lost productivity (U.S. Census Bureau 2008). This measure was converted into daily median personal income.

There are few extant estimates of the total number of days of lost productivity to a patient as a consequence of a respiratory illness treated at an ED. The effects of severe asthmatic attacks could incapacitate a patient for weeks or even months [National Heart, Lung, and Blood Institute (NHLBI) 2007]. Even so, Rodrigo et al. (2004) relied on a British study (Hoskins et al. 2000) that estimated that indirect costs (lost productivities) accounted for only 10% of the costs of asthma illnesses. Here we refer to the U.S. and British guidelines for the treatment of moderate asthma illnesses (British Thoracic Society 2008; NHLBI 2007), potentially requiring an ED visit. These guidelines suggest, on average, 1 day for treatment and up to 2 days for recuperation, for a total of 3 days of lost productivity per illness.

The population that is adversely affected by aerosolized brevetoxins is composed of Florida residents as well as tourists or longer-term visitors (snowbirds) from other states and other countries. The AHCA compiles data on the state of residence of patients visiting the SMH ED for respiratory ailments. We used the residential distribution of these patients to weight annual median personal income by state to determine a weighted average median personal income of $38,589. Three days of lost productivity therefore has an estimated value of approximately $335 (2008 dollars).

In Table 5, we calculated ranges of costs of illness for Sarasota County and for the Florida Gulf Coast. We used the predicted number of ED visits from model II in combination with the range of marginal costs of ED respiratory visits for UAD/bronchitis illnesses from the AHCA data. We estimated annual costs, 5-year costs, and capitalized costs using a discount rate of 3%. We chose this discount rate as reflective of the historical rate of return on a safe asset, such as medium-term government bonds. The latter two estimates imply that the low-, medium-, or high-bloom levels persist during the period of interest. We found that the capitalized costs of illness can range from $0.5 to $4 million in Sarasota County alone. We expect that the costs of illness should be much greater for the entire Florida Gulf Coast.

## Discussion

We investigated whether HABs can explain respiratory ED visits while controlling for relevant environmental factors that also may explain these visits. We found that *K. brevis* cell counts (lagged 1 week), low temperatures, a high incidence of influenza outbreaks, high pollen levels, and large numbers of tourist visits (including the snowbirds) all explained the number of respiratory-specific ED diagnoses. An individual’s ED visit also could be the result of multi day exposure to numerous, interacting conditions (e.g., aerosolized brevetoxins combined with environmental particulates, disease, local weather conditions) rather than the influence of one factor over a short time period. Statistical tests of our data failed to identify such interactions, however, suggesting an avenue for future research.

Analysis of our data revealed that ED visits for UAD and bronchitis are explained by the *K. brevis* cell count, but ED visits for pneumonia and asthma are not. However, Asai et al. (1982) observed that 80% of the 15 asthmatic patients exposed to red tide aerosols at the beach complained of asthma attacks, and, more recently, Fleming et al. (2005, 2007) found statistically significant changes in respiratory function in asthmatic residents of Sarasota after spending only 1 hr at a Florida beach during a red tide. One explanation for our finding is that asthmatics may be conditioned to recognize their symptoms, responding with preexisting prescriptions, over-the-counter medications, or outpatient doctor visits, instead of visits to the ED.

Estimates of the costs of illness are only one component of the total economic impact of *K. brevis* blooms along the Florida Gulf Coast. Notably, other costs of illness relating to illness severities and different effects, such as shellfish poisoning, are likely to exist. Additional costs may be associated with accessing primary care physicians, allergists, or pulmonologists, as well as prescriptions and over-the-counter medications. These data were unavailable for this study, however. Although there is no obvious evidence of mortalities as a consequence of respiratory disease, chronic respiratory problems may be triggered or exacerbated by aerosolized brevetoxins. Here, we did not attempt to estimate the costs of additional medical services required to treat chronic illness. Consequently, our cost-of-illness estimates should be considered conservative.

Further, we did not account for the non-market costs associated with pain and suffering when an individual experiences respiratory problems. When a bloom occurs, people often avoid visiting the beach; thus, additional non-market economic losses are associated with this behavior. Morgan et al. (2009) recently measured economic impacts in the coastal restaurant trade during HAB events and indicated that other tourism-related losses, such as declines in snowbird visits, could have broader impacts on the regional economy.

As the human migration to the coast of the Gulf of Mexico shows little evidence of abating, it will be necessary to consider alternative means of mitigating the public health and economic impacts of this natural hazard. It is unclear at this juncture whether human activities have contributed to the observed increase in frequency and intensity of *K. brevis* blooms (cf., Gilbert et al. 2005). Preliminary mitigation strategies involve risk communication (poison information center hotlines, signage, informational brochures and materials), natural resource management (shellfish bed closures), and nutrient controls (municipal fertilizer ordinances). All of these strategies entail costs.

## Conclusions

We found that the number of respiratory-specific ED diagnoses at SMH for UAD/bronchitis were correlated with lagged *K. brevis* cell counts, low air temperatures, influenza outbreaks, high pollen counts, and tourist visits. Extrapolating to Sarasota County, we estimated that the capitalized costs of illness for ED respiratory illnesses associated with *K. brevis* blooms in Sarasota County alone ranged from $0.5 to $4 million. The costs of illness of ED visits are a conservative estimate of the total economic impacts of *K. brevis* blooms in Sarasota County and for the Florida Gulf Coast. Because this is only a portion of the total economic impact of these events, it will become increasingly necessary to understand the full scale of the economic losses associated with *K. brevis* blooms in order to make rational choices about appropriate mitigation.

## Figures and Tables

**Table 1:** Descriptive statistics for model variables.

Variable	Description	Mean ± SD	Minimum	Maximum
lnED_tot_*_t_*	Natural logarithm of the daily average visits per week to SMH ED for respiratory ailments (visits)	2.40 ± 0.35	1.69	3.29
lnED_ub_*_t_*	Natural logarithm of the daily average visits per week to SMH ED for bronchitis and UAD (visits)	2.11 ± 0.37	1.35	3.15
HAB*_t_*_−1_	Prior week’s transformed *in situ* cell count (square root of HAB cells × 10^−3^)	8.11 ± 18.87	0.00	136.39
Temp*_t_*	Average weekly air temperature (°C)	23.08 ± 4.72	8.41	29.77
Temp*_t_*_−1_	Prior week’s average weekly air temperature (°C)	23.07 ± 4.72	8.41	29.77
Flu*_t_*	Weekly percentage of specimens testing positive for influenza in the CDC South Atlantic Region	6.34 ± 9.53	0.00	42.59
Pollen*_t_*	Weekly transformed total pollen count (square root of pollen grains/m^3^ air × 10^−2^)	1.10 ± 1.20	0.24	6.55
Tourist*_t_*	Estimated weekly transformed number of tourists to Sarasota County (persons × 10^−4^)	2.14 ± 0.38	1.30	2.98

**Table 2:** Parameter estimates for Yule-Walker autoregression models.

	Model I	Model II
Dependent variable	lnED_tot_	lnED_ub_
Intercept	2. 9169 (17.76)#	2.6328 (14.38)#
HAB*_t_*_−1_	0.0012 (1.81)*	0.0019 (2.54)#
Temp*_t_*	−0.0120 (−2.60)#	−0.0107 (−2.05)**
Temp*_t_*_−1_	−0.0241 (−5.06)#	−0.0277 (−5.17)#
Flu*_t_*	0.0096 (4.03)#	0.0103 (3.89)#
Pollen*_t_*	0.0286 (1.66)*	0.0319 (1.64)*
Tourist*_t_*	0.1003 (1.89)*	0.1161 (1.95)*
Observations (*n*)	261	261
DW	1.96	1.96
*R*^2^	0.76	0.74

DW, Durbin-Watson test statistic. *t*-Values are shown in parentheses.

* *p* < 0.1.

** *p* < 0.05.

# *p* < 0.01.

**Table 3:** Marginal effects and elasticities of model II variables (all effects and elasticities taken at the means of model variables).

	Marginal effects	Elasticities
HAB*_t_*_−1_	0.02	0.01
Temp*_t_*	−0.09	−0.24
Temp*_t_*_−1_	−0.23	−0.61
Flu*_t_*	0.09	0.06
Pollen*_t_*	0.26	0.03
Tourist*_t_*	0.96	0.24

**Table 4:** Sarasota County ED visits due to *K. Brevis* blooms (model II).

	Low	Medium	High
Annual average	39	76	218
5-Year estimate	197	376	1,090

**Table 5:** Annual cost-of-illness estimates for Sarasota County (2008 $, millions).^a^

	Low	Medium	High
Annual ($)	0.02–0.02	0.03–0.04	0.09–0.13
5-Year ($)	0.08–0.11	0.15–0.22	0.43–0.63
Capitalized [at 3% ($)]	0.52–0.76	1.00–1.45	2.86–4.18

a Using low, medium, and high predicted ED visits from model II for Sarasota County and the 25th and 75th quartiles of estimated marginal costs from the AHCA data for 2005.
